# Steady nutrient upwelling around a biological hotspot of the confluence between the quasi-stationary jet and the Oyashio in the western North Pacific

**DOI:** 10.1038/s41598-024-68214-z

**Published:** 2024-07-30

**Authors:** Itsuka Yabe, Shin-ichi Ito, Shigeho Kakehi, Takeyoshi Nagai, Jun Nishioka

**Affiliations:** 1 Atmosphere and Ocean Research Institute, The University of Tokyo, Kashiwa, Japan; 2 Tokyo University of Marine Science and Technology, Tokyo, Japan; 3 Japan Fisheries Research and Education Agency, Shiogama, Japan; 4 Pan-Okhotsk Research Center, Institute of Low Temperature Science, Hokkaido University, Sapporo, Japan

**Keywords:** Environmental sciences, Ocean sciences

## Abstract

The quasi-stationary jet, a branch of the Kuroshio Extension, transports warm saline water in the mixed water region of the western North Pacific. Around the subarctic front between the quasi-stationary jet and Oyashio and its downstream area is a biologically productive area including small pelagic fishes. However, how nutrient is supplied to the euphotic zone in this region remains elusive, especially into the quasi-stationary jet. Using high-resolution hydrography sections across the jet, we showed that Oyashio water isopycnally intrudes under the jet around 26.5–26.8 σ_θ_ and forms nutrient-rich intermediate water. Upwelling associated with ageostrophic secondary circulation across the front, caused by confluence, uplifts the intermediate water. A local nitrate maximum was also identified inside the jet by the hydrographic observation. Upwelling has been suggested as a precondition for nutrient supply from nutrient-rich intermediate water to the jet through water mixing which potentially sustains high biological production in the downstream.

## Introduction

The frontal region in oceans, especially where two ocean currents converge, is often an active upwelling and downwelling area that promotes vertical exchange of inorganic and organic matter. Ageostrophic secondary circulation across this region forms these vertical motions^1^. Research based on hydrographic surveys and numerical simulations has been conducted for major ocean currents worldwide, such as the Antarctic Circumpolar Current, Gulf Stream, and Kuroshio Extension (KE), to estimate the ageostrophic secondary circulation that causes nutrient mixing, followed by increases in primary production^2–5^. Nagai et al.^3^ indicated that ageostrophic flows induced by confluence and friction supply nutrient and enhance primary production on the warm side of the front. Kouketsu et al.^6^ observed upwelling (downwelling) from the trough (crest) to the crest (trough) at the surface frontal wave, and suggested that this wave led to the isopycnal intrusion of the intermediate water and mixing across the front owing to baroclinic instability.

**Figure 1:**
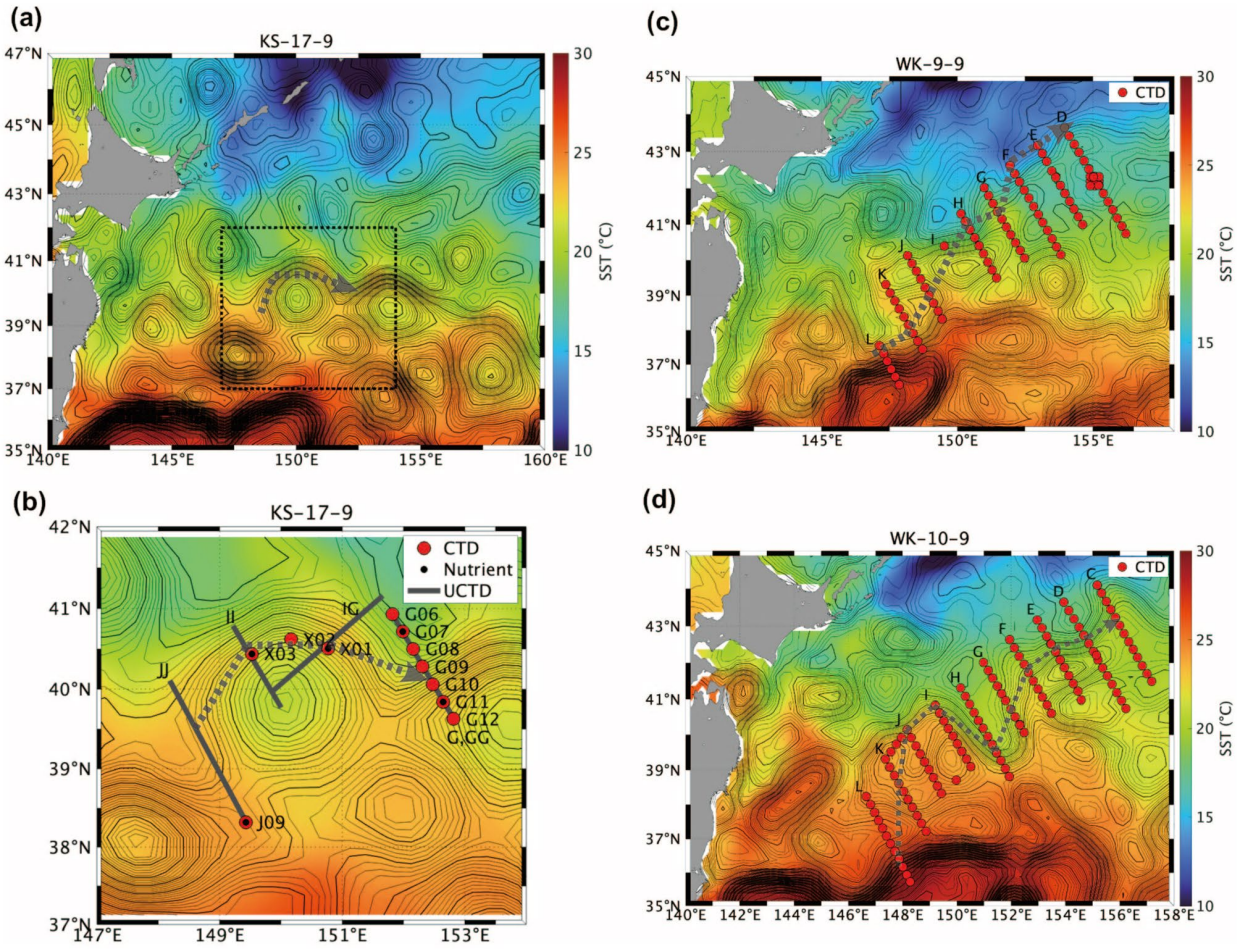
SST (°C, color) and sea surface height (SSH, m, contour) during cruises in (**a**) 2017 (KS-17-9), (**c**) 2009 (WK-9-9), and (**d**) 2010 (WK-10-9). (**b**) Zoom of the box in (**a**). Contour intervals are 0.01 and 0.05 m for thin and thick lines, respectively. In each panel, locations of conductivity-temperature-depth (CTD) stations (red circles), bottle samples (black circles), and underway CTD (U-CTD) measurement lines (thick gray lines) are shown. Gray dotted lines illustrate the QSJ pathway.

The subarctic front (SAF) in the western North Pacific is the temperature and salinity front and the northern boundary of the mixed water region (MWR) between Oyashio and Kuroshio^7,8^. Quasi-stationary jets are a branch of KE that transport the heat and salt of the Kuroshio water (subtropical water) into the MWR and subarctic regions^9–11^. A robust horizontal gradient of sea surface temperature (SST) exists along SAF, which is the confluence area of the return flow of Oyashio and quasi-stationary jets^9,12,13^. Herein, we focused on the western jet of the two quasi-stationary jets (hereinafter called QSJ), which is located at approximately 150–155° E and is bordered by the return flow of Oyashio (Fig. 1).

MWR, especially around the QSJ pathway, is a high productivity area in the western North Pacific^14^. Continuous nutrient supply to the euphotic zone is essential to support high primary production in the MWR around QSJ. In the surface layer shallower than 100 m, Oyashio water mixed with Kuroshio water may be responsible for supplying nutrient to the QSJ surface^15^. Indeed, the main zooplankton species around QSJ were not only subtropical species but also large subarctic species^16^, implying the importance of Oyashio water (subarctic water) for nutrient supply to the QSJ. However, since nutrient generally become enriched with depth, mechanisms for transporting nutrients upward are needed. The isopycnal nitrate flux at the density range 26.6–27.4 σ_θ_ from the intermediate water of Oyashio to the MWR is estimated at approximately 110 kmol s^−1^; although this flux exhibits a maximum at approximately 250 m depth^17^, which is deeper than the euphotic layer, the nutrient injected to the shallower depths are more susceptible to surface mixing during winter. However, whether and how subsurface nutrient is supplied to the euphotic zone in other seasons remains unclear because in situ observations are lacking.

The MWR is on the migration routes from subtropical spawning grounds to subarctic feeding grounds for many pelagic fishes, such as the Pacific saury *Cololabis saira*, and Japanese anchovy *Engraulis japonicus*^18^. In addition to prey density, seawater temperature is a major component affecting their growth and survival^19^. Although the warm temperature in QSJ positively affects their growth, the nutrient concentration is lower than that of the Oyashio water. Thus, nutrient supply to QSJ water is a key to support the high productivity of small pelagic fishes following primary production^15,16,20^.

Herein, we analyzed multi-year hydrographic data on physical water properties and nutrient, including high vertical-resolution nitrate data across the QSJ. Particularly, we aimed to clarify the process of continuous nutrient supply to the QSJ water and elucidate where the nutrient supply in the upstream regions occurs.

## Results

### Hydrographic structure

**Figure 2:**
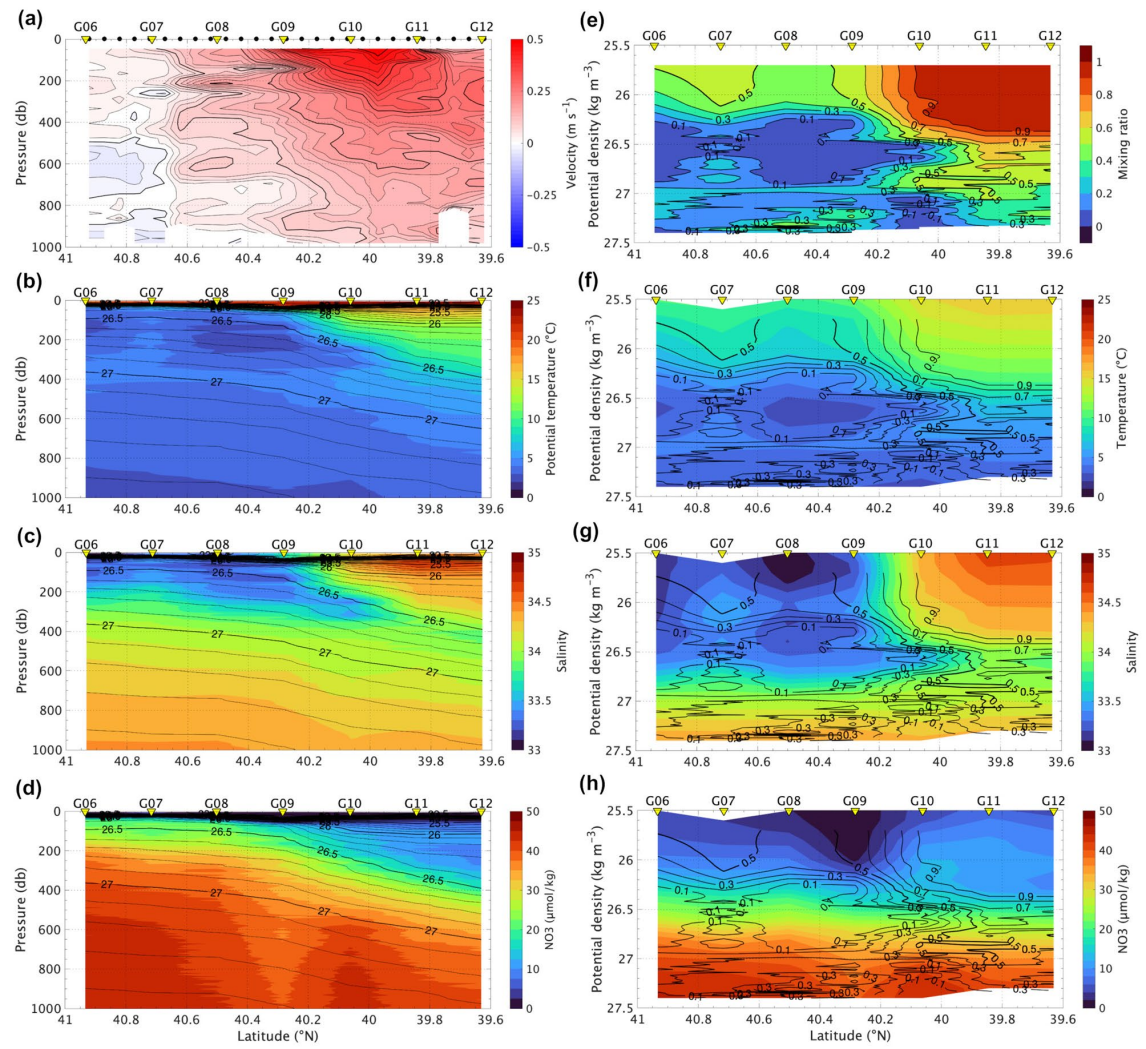
(**a**) Current velocity measured by ship-mounted ADCP (m s^−1^), (**b**, **f**) potential temperature (°C), (**c**, **g**) salinity, (**d**, **h**) nitrate (μmol kg^−1^), and (**e**) mixing ratio along G-line in KS-17-9. Velocity indicates approximately northeastward direction across the G-line. The mixing ratio of 1.0 indicates pure Kuroshio water. Vertical coordinate of (a, b, c, d) is a function of pressure (db), and of (**e**, **f**, **g**, **h**) is a function of﻿ σ_θ_﻿ (kg m^−3^). Isolines in (**b**, **c**, **d**) indicate σ_θ_﻿, whose intervals are 0.1 and 0.5 kg m^−3^ for thin and thick lines, respectively. Isolines in (**f**, **g**, **h**) indicate the mixing ratio, whose intervals are 0.1 and 0.5 for thin and thick lines, respectively.

Current axis associated with the QSJ exists around 40° N, whose current velocity measured by ship-mounted acoustic Doppler current profiler (ADCP) exceeded 0.5 m s^−1^ at the surface on the vertical section of CTD and ADCP measurements (hereinafter called G-line) for the cruise in 2017 (KS-17-9). The northeastward current has deep structure with the velocity of 0.1 m s^−1^ at 1000 m depth (Fig. 2a). At station G12, another peak in the velocity was observed at 200 m depth, corresponding to an anti-cyclonic eddy (ACE) that exists in the southern part of the G-line (Fig. 1a and b).

Warm (> 10 °C) and saline (> 34.1) QSJ water as defined in the Methods existed at the surface layer shallower than 26.5 σ_θ_ at G10–G12, while cold (< 5 °C) and low-salinity (< 33.8) Oyashio water was distributed to the north of G9 (Fig. 2b and c). The sharp temperature and salinity front associated with the QSJ was approximately 40.2° N between 0 and 100 m, and the front corresponded to the northern end of QSJ. Velocity exhibited two maxima of the QSJ and ACE (Fig. 2a), but water masses based on temperature and salinity could not classify these two velocity features (Fig. 2b and c). The isopycnal lines deepened to the south, and an isopycnal intrusion of the cold and low-salinity water under the QSJ water was identified around 26.5–26.8 σ_θ_ (Fig. 2b and c).

**Figure 3:**
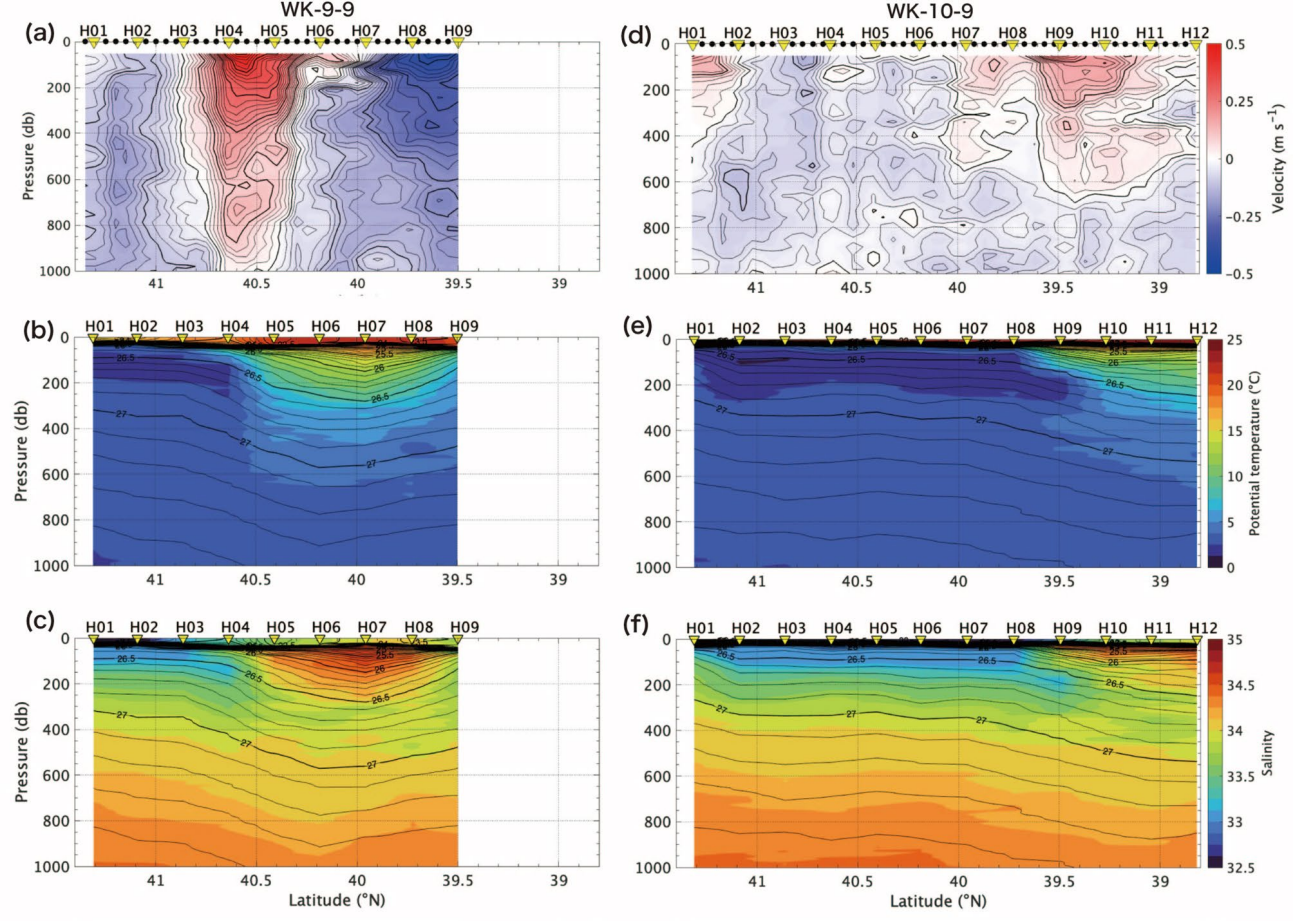
(**a**, **d**) Current velocity measured by ADCP (m s^−1^), (**b**, **e**) potential temperature (°C), and (**c**, **f**) salinity along H-line in WK-9-9 and WK-10-9. Details of each figure is same as Fig. 2.

Figure 3 shows the hydrographic structures on the H-line during the cruise in 2009 (WK-9-9), and in 2010 (WK-10-9). We select the H-line, because the isopycnal intrusion of the cold and low-salinity water under the QSJ was the strongest on this line in both years. The front between the Oyashio and the QSJ was approximately 40.6° N between H04 and H05 in 2009, and 39.6° N between H08 and H09 in 2010. Latitudinal position of the front was affected by eddies in the MWR, and meandering of the northeastward return flow of the Oyashio (Fig. 1), as mentioned by the Isoguchi et al.^9^. Local minimum of temperature and salinity under the QSJ water and the isopycnal intrusion of the cold and low-salinity water under the QSJ water was identified at the same density range as KS-17-9 around 26.5–26.8 σ_θ_, although the position of the front varied (Fig. 2). This intrusion is suggested to be the origin of the North Pacific intermediate water in the subtropical gyre^21^.

The nitrate concentration was < 10 μmol kg^−1^ south of the surface front at 50 m depth (~ 40.2° N), while it was > 20 μmol kg^−1^ north of the front (~ 40.2° N) (Fig. 2d). In both areas, the concentrations gradually increased with depth. In areas deeper than 600 m, a local nitrate maximum (over 40 μmol kg^−1^) was observed at G10, corresponding to the QSJ flow axis.

### Isopycnal variation of the mixing ratio across the QSJ

The mixing ratio between the Kuroshio and Oyashio water was > 0.8 in warm and saline QSJ waters, corresponding to nearly pure Kuroshio water (Fig. 2e). Under QSJ waters, the mixing ratio gradually decreased with depth. North of the QSJ, the mixing ratio was < 0.3 (Oyashio water defined in the Methods). The mixing ratio showed a local minimum in the middle layer around 26.5 σ_θ_. Between G9 and G11, the Oyashio water intruded under the QSJ water along the isopycnal range around 26.5–26.8 σ_θ_ (Fig. 2e). Additionally, the local minimum of the mixing ratio was at G10 at density > 27.0 σ_θ_. Above the Oyashio water, the mixing ratio was approximately 0.5 around 25.7–26.0 σ_θ_, and the Kuroshio and Oyashio waters were mixed equally.

The nitrate concentration gradually increased with density and exceeded 30 μmol kg^−1^ in the layer with density > 26.7 σ_θ_ (Fig. 2h). The local nitrate maximum was observed at G10 at a density > 27.0 σ_θ_ (Fig. 2h), whose location corresponded to the minimum of the mixing ratio (Fig. 2e). Interestingly, anomalously low nitrate concentrations on surfaces with < 26.25 σ_θ_ density were near G09 at the QSJ northern edge; that is, nitrate concentrations were elevated on both sides of the front across the QSJ above the low-salinity intrusion.

### Spatial variation of the mixing ratio along the QSJ

We suppose that the water mass mixing and supply of nutrient into the QSJ at G10 at a density around 26.2 σ_θ_ had already occurred upstream of the G-line and flowed into the line. We examined where mixing occurred between the QSJ and Oyashio. We only had temperature and salinity values upstream of the G-line from U-CTD observations and no nitrate values. We estimated the mixing area using the relationship between the nitrate concentration, salinity, and mixing ratio.

**Figure 4:**
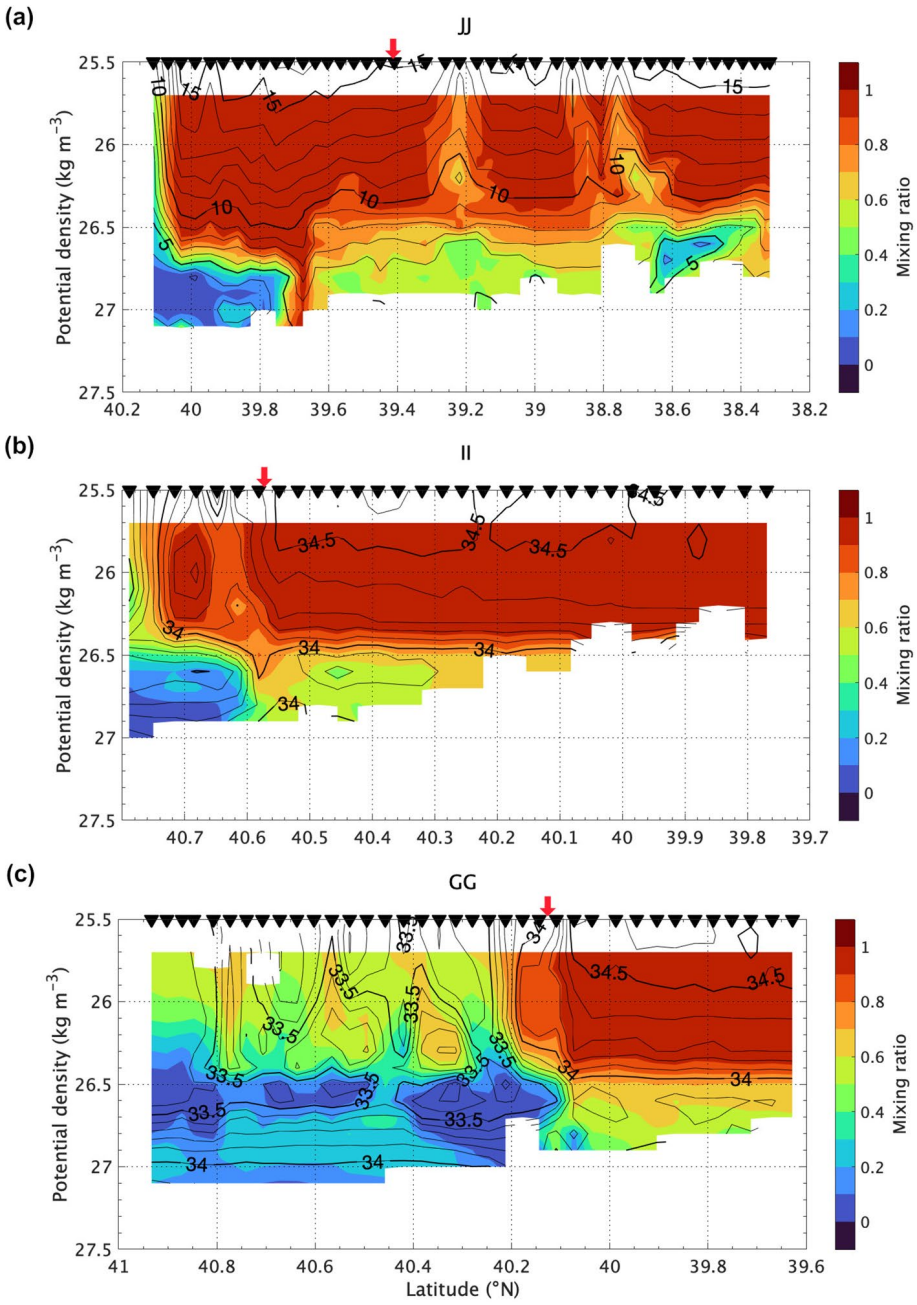
The mixing ratio (color) and salinity (contour) along (**a**) JJ-line, (**b**) II-line, and (**c**) GG-line as a function of σ_θ_. Inverted triangles above each panel indicate where U-CTD were deployed. Red arrows denote the location of surface velocity maxima across each line.

The upstream observation II and JJ lines across the ACE were located around 40° N and 150° E (Fig. 1b), and the QSJ flow axis was located in the northern part (Fig. 4). The mixing ratio was > 0.9 around the QSJ at density range < 26.5 σ_θ_ in both lines. The Oyashio water, with a mixing ratio of < 0.2 intruded from the northside of the section under the Kuroshio water. The mixing ratio around the QSJ (~ 39.4° N) on the JJ-line was approximately 1.0 (Fig. 4a), whereas that on the II-line decreased to 0.7–0.9 (~ 40.6° N) (Fig. 4b), suggesting mixing with the Oyashio water. The mixing ratio did not significantly differ between II and GG lines (Fig. 4b and c). The isopycnal intrusion of the Oyashio water under the QSJ advanced from GG to JJ.

**Figure 5:**
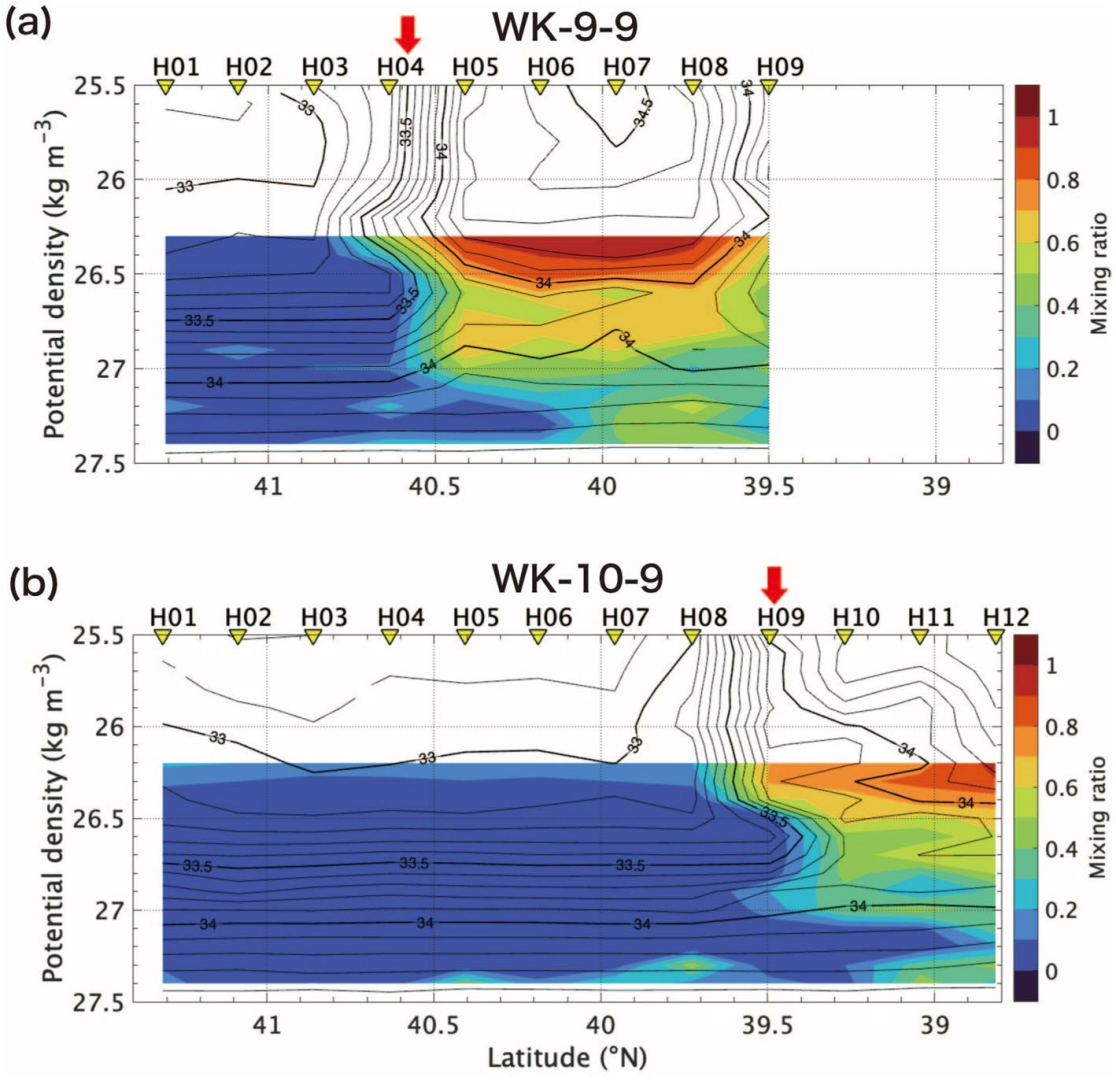
The mixing ratio (color) and salinity (contour) along H-line during (**a**) WK-9-9, and (**b**) WK-10-9 as a function of σ_θ_. Inverted triangles above each panel indicate where CTD were deployed. Red arrows denote the location of surface velocity maxima across each line.

**Figure 6:**
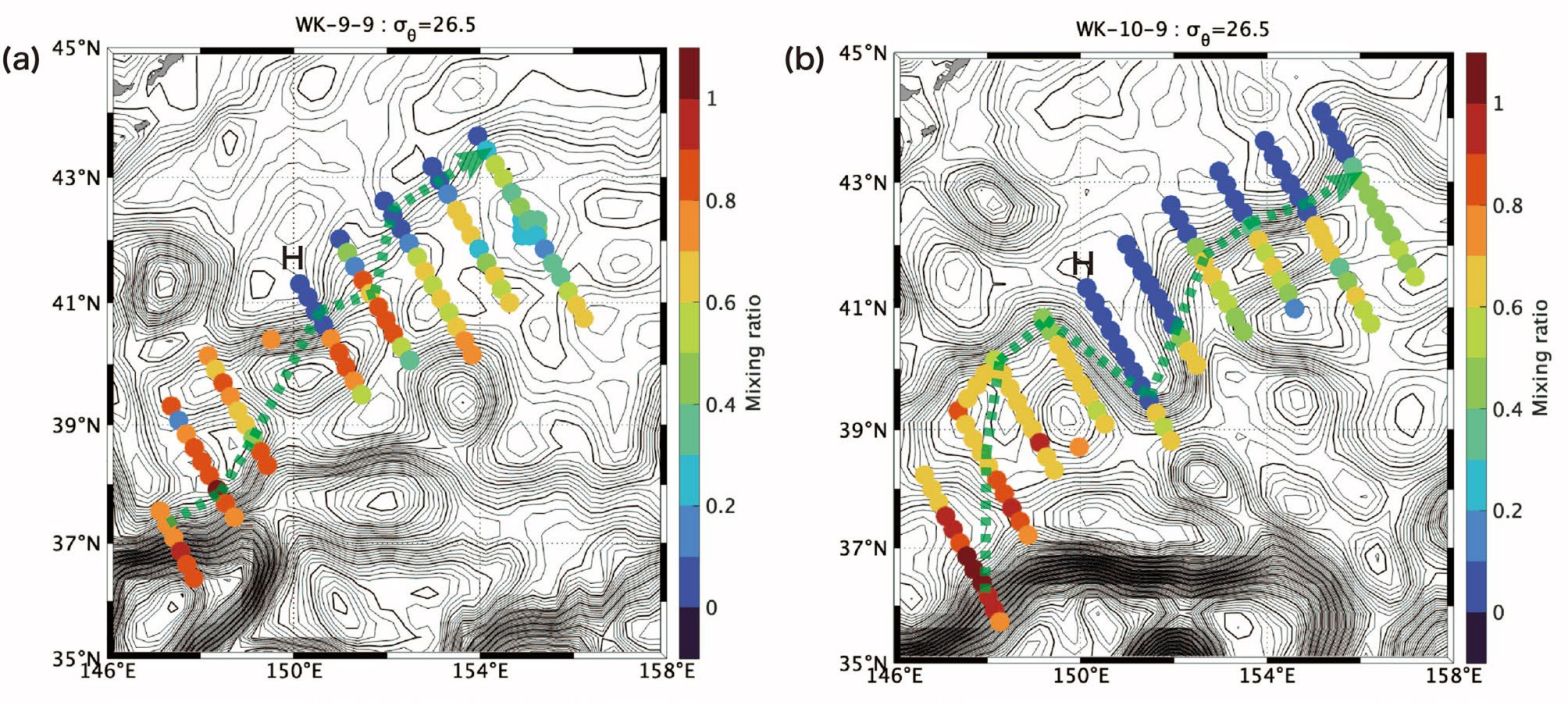
Horizontal map of the mixing ratio along σ_θ_ is 26.5 during (**a**) WK-9-9, and (**b**) WK-10-9. Isolines show the SSH (m) during each cruise, whose intervals were 0.01 and 0.05 m for thin and thick lines, respectively.

Figure 5 shows the salinity and the mixing ratio as a function of σ_θ_ along the H-line during WK-9-9, and WK-10-9. The mixing ratio across the front between the QSJ and the Oyashio shows similar pattern to the distribution in KS-17-9: the Oyashio water intruded under the QSJ water along the isopycnal range around 26.5–26.8 σ_θ_ with the mixing ratio of about 0.5 in addition to the local minimum of the mixing ratio at density > 27.0 σ_θ_. (Fig. 2e, g). The isopycnal intrusion under the QSJ water was stronger in 2010 than in 2009 (Fig. 5). Figure 6 shows horizontal distribution of the mixing ratio along 26.5 σ_θ_. According to Fig. 6, the mixing ratio decreased from upstream to downstream of the front, as indicated by Kakehi et al.^15^. The Oyashio water intruded southward on the H-line, and the front judged from the SSH meandered southward (Fig. 6). The isopycnal intrusion was also observed at the density greater than 27.0 σ_θ_.

**Figure 7:**
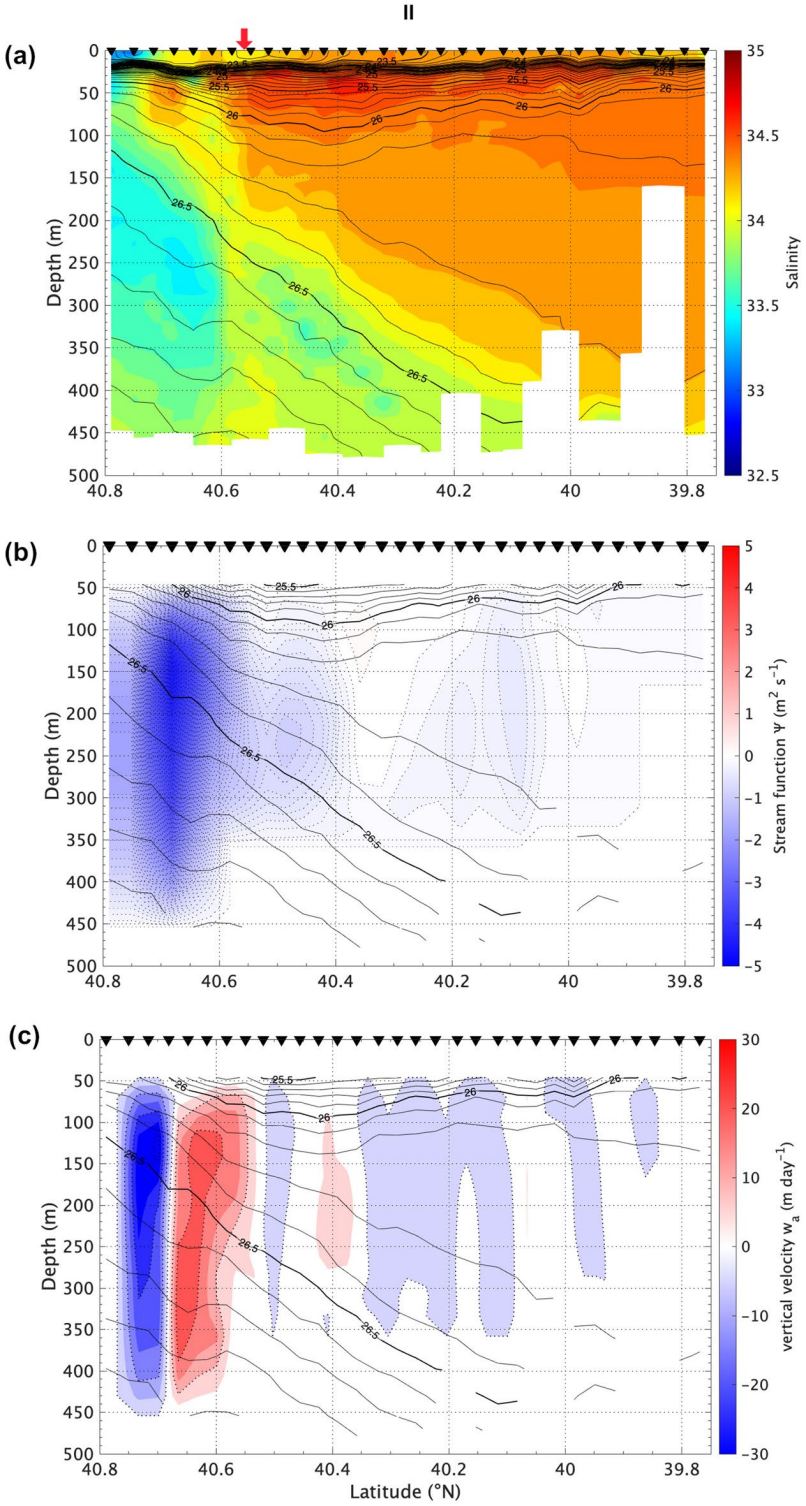
(**a**) Salinity, (**b**) stream function of ageostrophic current, and (**c**) vertical velocity (positive in upward) as a function of depth. Solid isolines indicate σ_θ_, whose intervals are 0.1 and 0.5 kg m^−3^ for thin and thick lines, respectively. Intervals of dotted isolines in (**b**) and (**c**) are 0.1 m^2^ s^−1^ and 10 m d^−1^, respectively. Inversed triangles and red arrows are the same as those in Fig﻿. 5.

A local minimum of mixing ratio (ca. 0.8) was distributed across density layers over 25.7–26.3 σ_θ_ around 40.6° N (Fig. 4b), suggesting that diapycnal mixing occurred on II-line. Additionally, a local minimum of salinity existed at 25.7–26.3 σ_θ_ between 39.0 and 39.5°N in 2010 (Fig. 5b). According to Fig. 6, the mixing ratio does not decrease with the isopycnal mixing from upstream side of the QSJ. Vertical or horizontal mixing with Oyashio water across the isopycnal surface is required to reduce the mixing ratio in the QSJ water. The vertical section of salinity on II-line implied that low-salinity Oyashio water intruded into QSJ water at < 200 m depth at approximately 40.6° N (Fig. 7a).

### Upwelling by ageostrophic vertical circulation

The barotropic deformation rate α that forces the omega equation (see Methods) was estimated to be O(10^−6^ s^−1^) in the MWR, and it locally exceeded 1.0 × 10^−5^ s^−1^ along the QSJ. It was almost comparable with the values along the KE^22^. The estimated ageostrophic secondary flow indicated that a counterclockwise vertical circulation with a vertical scale of almost 400 m existed north of the QSJ axis, and was associated with upwelling/downwelling on the southside/northside of the circulation diagnosed on II-line (Fig. 7b and c). The vertical scale and velocity of the circulation were the largest among the four lines (figures not shown). The maximum upwelling velocity was approximately 30 m d^−1^ at 150 m depth. The vertical current moved the isopycnal surfaces vertically toward the flow direction, which were distorted north of the QSJ and downward under the QSJ at 250–450 m depth. This suggested that the distortion direction was consistent with the ageostrophic currents. The along-isopycnal intrusion/subduction of low-salinity water roughly coincided with the position of the diagnosed downwelling, where the combined flows induced by the ageostrophic secondary circulation and confluence moved water nearly along the isopycnal direction.

## Discussion

**Figure 8:**
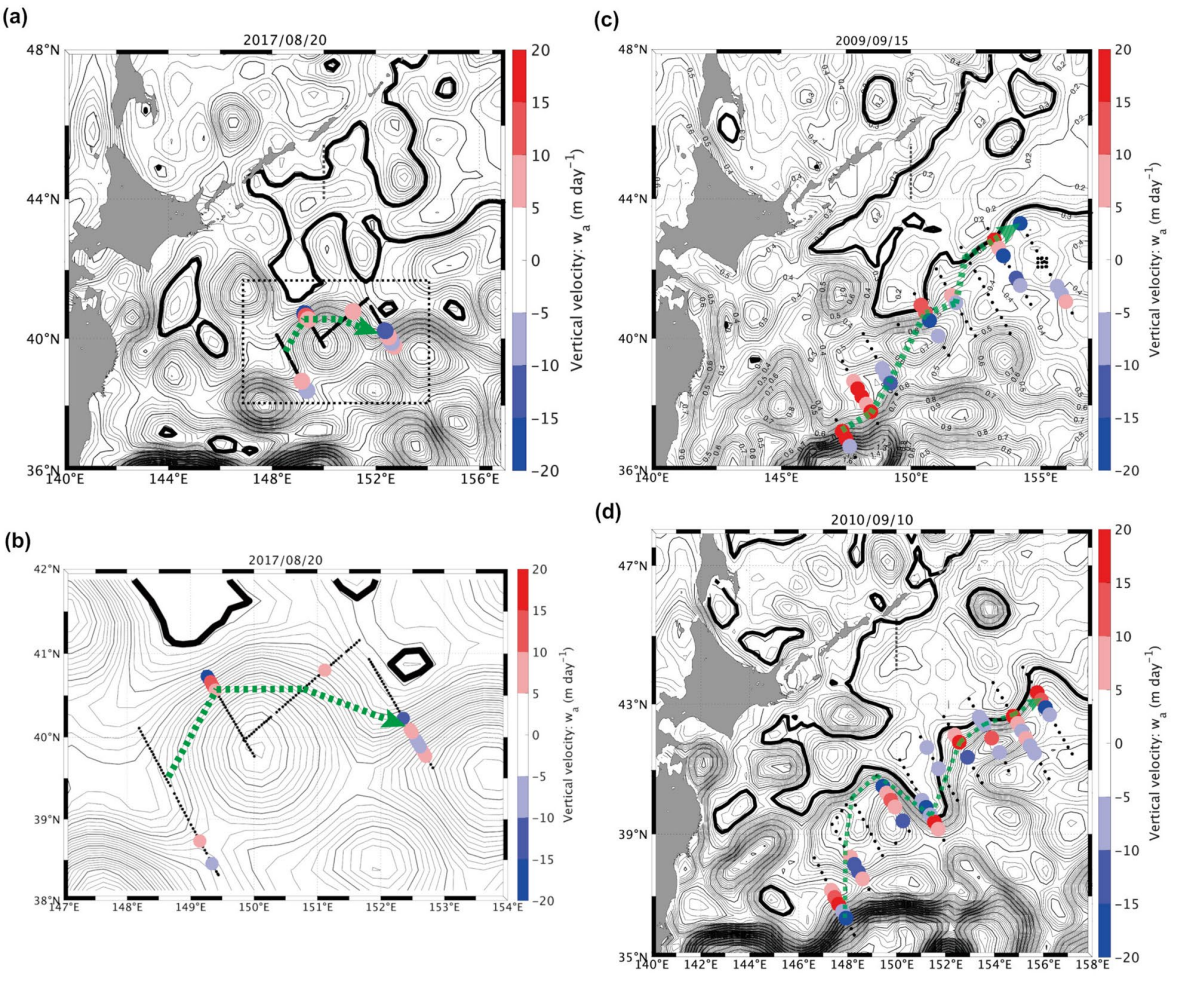
Vertical averaged upwelling/downwelling velocity based on secondary circulation in (**a**) KS-17-9, (**c**) WK-9-9, and (**d**) WK-10-9. (**b**) Zoom of the indicated box in (**a**). Stations, where the absolute vertical velocity was > 5 m s^−1^, were only marked. Isolines show the SSH (m) during each cruise, whose intervals were 0.01 and 0.05 m for thin and thick lines, respectively. Bold black isoline indicates the rough pathway of Oyashio, assuming SSH value at the current axis along the black dotted line. Green dotted lines illustrate the QSJ pathway, connecting the flow axis of each observation line.

We found two local nitrate maxima: around 100–150 m depth (26.0 σ_θ_) inside the QSJ, and below the QSJ flow axis deeper than 600 m (27.0 σ_θ_) (Fig. 2d and h). The ageostrophic vertical circulation suggested that the upwelling lifted the deep nutrient-rich water, causing nutricline around 26.5 σ_θ_ under the QSJ at II-line (Figs. 4b and 7). According to the SSH isolines (Fig. 8a), QSJ meandered northward around II-line and approached the subarctic gyre. Nakano et al.^23^ indicated that SSH isolines reproduce the KE, SAF, and subarctic boundary pathways in the North Pacific. We determined the SSH value of the Oyashio current axis off the Kuril Islands, which roughly indicated the Oyashio pathway (bold black lines in Fig. 8). The position of QSJ was confirmed by hydrographic observations (green dotted lines in Fig. 8). The frontal structure between the QSJ and Oyashio became stronger around the approaching point of the two currents. The cross-sectional distribution of upwelling and downwelling revealed that maximum upwelling occurred around the approaching point between the two currents (40.7° N and 149° E in II-line) (Fig. 8a and b).

QSJ transports warm water northeastward, with interannual variations affected by mesoscale eddies (Fig. 1). The maximum vertical mean upwelling velocity exceeded 15 and 20 m d^−1^ in 2017, and 2009 and 2010, respectively (Fig. 8). Additionally, maximum upwelling velocities were observed around the confluence between QSJ and Oyashio for all three years. Based on these results, we hypothesized that upwelling occurs under QSJ at the confluence of QSJ and Oyashio. Thus, nutrient supply to the euphotic zone in QSJ may have occurred around the upwelling area, as identified in 2017. This upwelling is believed to enhance the biological production downstream of QSJ and contribute to the formation of a biological hotspot in the offshore area, including small pelagic fish aggregates. Although the current magnitude of the QSJ is relatively small compared with the other ocean currents around the MWR, e.g., the Kuroshio, and the Oyashio, the upwelling velocities estimated in this study were comparable to the upwelling velocity of 10–40 m d^−1^ in the Gulf Stream^24^, 10–100 m d^−1^ in the Kuroshio Extension^25^, and O(10 m d^−1^) in the Azores Front^22^. In addition, there was a possibility that the vertical scale of the front was underestimated on the II-line, because the UCTD measurements could reach only down to 500 m depth. Deeper vertical circulation with a deeper frontal structure would increase the estimated upwelling and deepen the upwelling cell, leading to more nutrient upwelling.

Using numerical simulation, Nagai et al.^3^ identified that vertical mixing and large-scale deformation work together to induce ageostrophic circulation, playing essential roles in nutrient supply to the euphotic zone and associated primary production. Additionally, the combination of frontogenesis and ageostrophic circulation creates submesoscale structures that affect phytoplankton growth^5^. Vertical mixing along the frontal structure of the QSJ is stronger in areas surrounding the MWR^26,27^. Further studies are required to clarify the mixing process of waters, because this vertical current does not directly cause mixing. Patchy low-salinity water with vertical extents of several tens of meters suggested the importance of mixing associated with submesoscale flows (Fig. 7a). However, the role of diapycnal mixing processes and adiabatic vertical exchanges remains unclear and warrants further study.

## Conclusions

QSJ, a branch of KE, flows northeastward and transports warm, saline Kuroshio waters along the SAF in the western North Pacific. Nitrate concentration was found to increase in the QSJ, where the region of good feeding and fishery grounds for small pelagic fishes, owing to the warm environment and prey abundance. Nitrate-rich Oyashio water intruded isopycnally under the QSJ water (Kuroshio water). Strong upwelling due to ageostrophic secondary circulation was diagnosed which could have induced nutricline uplift on the warm side of the front around the confluence. Since quasi-steady flows of the QSJ and Oyashio continuously form confluence in the MWR of the western North Pacific, nutrient is constantly supplied from the Oyashio waters to the QSJ.

Nutrient is supplied to the euphotic zone at the confluence of the two currents, with ageostrophic secondary circulation across the frontal structure between the currents. A steady nutrient supply to the euphotic zone with moderately strong currents seems to contribute the formation of the biological hotspots near the QSJ. As moderately strong currents associated with the mesoscale structures are ubiquitous features in the oceans, similar nutrient supply mechanisms, that lead to biological productivity, need to be investigated in various oceans in the future.

## Methods

### Hydrographic observation

Temperature, salinity, current, and nutrient data were collected from the R/V Wakataka-Maru of the Japan Fisheries Research and Education Agency (FRA) in 2009 (WK-9-9 cruise) and 2010 (WK-10-9 cruise), and the R/V Shinsei-Maru of the Japan Agency for Marine-Earth Science and Technology in 2017 (KS-17-9 cruise). Multiple sections were created across the QSJ flow axis (Fig. 1). Temperature and salinity profiles were observed using a CTD (Sea-Bird Scientific) and a U-CTD (Teledyne Ocean Science). Water samples were collected at the CTD stations to measure the nutrient concentrations at sampling depths of 10, 50, 100, 150, 200, 300, 500, 800, 1000, 1250, and 1500 m. Salinity were calibrated using the water sample salinities. Nitrate concentration was measured at high vertical resolution using a nitrate sensor (ISUS; Sea-Bird Scientific) attached to the CTD during KS-17-9. We converted the voltage into concentration (μmol kg^−1^) based on linear regression using the bottle sample data (y = 27.96x − 12.56, correlation coefficient r = 0.9865, *p* < 0.01, N = 75). A ship-mounted ADCP (Ocean Observer, 38 kHz) continuously measured the horizontal current velocity during all three cruises. The ADCP current velocity was measured in 50 layers every 24 m for 1 min. We eliminated low-quality data with a percentage of good < 80% and a correlation greater than 64 count. The systematic error due to the misalignment angles between the transducer and gyrocompass was calibrated based on the method proposed by Joyce^28^.

### Satellite data

We used the absolute SSH and geostrophic surface current from satellite altimeter data distributed by the Copernicus Marine Service (10.48670/moi-00148). Additionally, merged satellite and in situ global daily SST data provided by the Japan Meteorological Agency (JMA, https://ds.data.jma.go.jp/gmd/goos/data/pub/JMA-product/), were used to examine the horizontal distribution of the QSJ and mesoscale eddies.

### Mixing ratio

The main mixing process between the Kuroshio and Oyashio waters in the MWR is known as isopycnal mixing^21^; accordingly, the mixing ratio assuming isopycnal mixing between the pure Kuroshio and Oyashio waters was calculated as the average of the temperature- and salinity-based mixing ratios, following the method of Shimizu et al.^29,30^. Data obtained from the R/V Keifu-Maru of the JMA in January 2009, and 2017, and R/V Ryofu-Maru in January 2010 of the JMA (https://www.data.jma.go.jp/gmd/kaiyou/db/vessel_obs/data-report/html/ship/ship_e.php) and the R/V Hokko-Maru of the FRA in January 2009, 2010, and 2017 (https://ocean.fra.go.jp/a-line/a-line_ctd.html) were used to define the temperature and salinity profiles of pure Kuroshio and Oyashio waters, respectively. Kawai^31^ indicated that the isotherm of 5 °C at 100 m depth was defined as the Oyashio Front. We averaged profiles with temperature less than 5 °C at 100 m depth to determine the pure Oyashio water. According to the SSH and geostrophic surface current, we picked up profiles inside the Kuroshio and these profiles were averaged to determine the pure Kuroshio water. The mixing ratio during KS-17-9, WK-9-9, WK-10-9 was respectively calculated below 25.7 σ_θ_, 26.3 σ_θ_, and 26.2 σ_θ_. Density range calculating the mixing ratio was different because the density around the surface was different in each cruise. In this study, warm (> 10 °C) and saline (> 34.1) water originating from the KE was defined as the QSJ water which corresponded to the mixing ratio was > 0.8 (Fig. 2). Cold (< 5 °C) and low-salinity (< 33.8) water with the mixing ratio was < 0.3 originating from the subarctic gyre was referred to as Oyashio water (Fig. 2).

### Ageostrophic vertical circulation across the front

To diagnose the upwelling and downwelling associated with ageostrophic secondary circulation across the front^1^, we used the following quasi-geostrophic omega equation^24^:

1 $${f}^{2}\frac{{\partial }^{2}\psi }{{\partial z}^{2}}+{N}^{2}\frac{{\partial }^{2}\psi }{{\partial x}^{2}}=2\left(\frac{\partial {u}_{g}}{\partial x}\frac{\partial b}{\partial x}+\frac{\partial {v}_{g}}{\partial x}\frac{\partial b}{\partial y}\right),$$

where x and y are the cross and along-stream coordinates, f is the Coriolis parameter, N is the buoyancy frequency, $\psi$ (${u}_{a}=\partial \psi /\partial z, {w}_{a}=-\partial \psi /\partial x$) is the stream function, $({u}_{g}, {v}_{g})$ is the geostrophic velocity, b is the buoyancy ($=-g\rho /{\rho }_{0}$, g is the gravity acceleration, $\rho$ is the density, and ${\rho }_{0}$ is the reference density), and $({u}_{a}, {v}_{a})$ is the ageostrophic velocity. After considering 60 min running mean for the ADCP data, we assumed the current velocity measured by the ADCP $({u}_{s}, {v}_{s})$ to be the geostrophic velocity while using Eq. (1). The along-stream gradient of the buoyancy term (second term) on the right-hand side of Eq. (1) is assumed to be much weaker than the cross-stream gradient; therefore, we only considered the first term. To estimate the vertical velocity, we set boundary condition ${w}_{a}$ = 0 at 500 m (because the U-CTD data covers 500 m), $\psi$ = 0 at $x$ = 0 and the end of the section. A barotropic deformation rate α indicates tendencies of frontogenesis and frontolysis^22,24^, and it is explained as:

2 $$\alpha ={\left[{\frac{1}{4}\left(\frac{\partial {v}_{g}}{\partial x}+\frac{\partial {u}_{g}}{\partial y}\right)}^{2}+{\left(\frac{\partial {u}_{g}}{\partial x}\right)}^{2}\right]}^\frac{1}{2}.$$

## Data Availability

Publicly available datasets were analyzed in this study. These data are available at https://www.jodc.go.jp/jodcweb/JDOSS/specific_data/20180031/20180031.html, 10.48670/moi-00148, https://ds.data.jma.go.jp/gmd/goos/data/pub/JMA-product/, https://www.data.jma.go.jp/gmd/kaiyou/db/vessel_obs/data-report/html/ship/ship_e.php/, https://ocean.fra.go.jp/a-line/a-line_ctd.html. Other physical and chemical data obtained from the survey used in the study are available on GitHub (data archiving is underway). Other datasets analysed during the current study are available from the corresponding author on reasonable request. MATLAB was used in generating all the figures.
